# Complete Genome Sequence of an Avian Bornavirus Isolated from a Healthy Canadian Goose (*Branta canadensis*)

**DOI:** 10.1128/genomeA.00839-13

**Published:** 2013-10-24

**Authors:** Jianhua Guo, John Baroch, Adam Randall, Ian Tizard

**Affiliations:** Department of Pathobiology, College of Veterinary Medicine and Biomedical Science, Texas A&M University, College Station, Texas, USAa; National Wildlife Research Center, WildlifeServices/APHIS/USDA, Fort Collins, Colorado, USAb; USDA, APHIS Wildlife Services, Pittstown, New Jersey, USAc

## Abstract

A recent survey among wild birds demonstrated the presence of a unique genotype of avian bornavirus (ABV) in wild geese and swans in North America. Here, we report the first complete genome sequence of an avian bornavirus of the goose genotype.

## GENOME ANNOUNCEMENT

Avian bornavirus (ABV) is the causative agent of proventricular dilatation disease, a fatal neurologic and gastrointestinal disease in psittacine and other birds (1, 2). The genomic sequences of different ABV isolates are much more variable than those of Borna disease virus (BDV) isolates (3). Recent surveys have demonstrated that a unique genotype of ABV also infects apparently healthy geese and swans across North America (4–6). Under some circumstances, it has been reported to cause lethal neurological disease in Canadian geese and trumpeter swans (7, 8). The viruses that infect geese and swans are apparently from a single genotype. We report here on the complete genomic sequence of ABV isolated from the brain of a healthy Canadian goose (*Branta canadensis*), collected in Union County, New Jersey, on 14 February 2011.

In this study, an ABV-positive goose brain sample was initially identified using a reverse transcriptase PCR (RT-PCR) employing primers targeting the M gene, as described previously (5, 6). A brain sample from this bird was then used to inoculate primary duck embryo fibroblasts (DEF). Following 3 serial passages, ABV growth was detected using RT-PCR. Two PCR products, approximately 3 and 6 kb in size, were cloned into the pCRTM4-TOPO vector and sequenced using a primer-walking approach by the Gene Technology Lab of Texas A&M University. The sequences were assembled with Sequencher 4.1, and phylogenetic analysis was performed using MEGA5.2.

The isolated genome from strain ABV-062_CG_ comprises 9,006 nucleotides. Its organization is very similar to that of BDVs and other published genotypes of ABV. An analysis of the intergenic region between the N and X genes revealed that this region varies greatly in length among BDV and ABV genotypes. Within this region, BDV has a short upstream open reading frame (uORF) coding for 8 or 9 amino acids that regulate the translation of the X protein (9). A similar uORF is present in ABV-062_CG_ but not in ABV1, ABV2, or ABV4. This suggests that ABV-062_CG_ might have a closer evolutionary relationship with BDVs than with other ABV genotypes. Although ABV isolates from canaries (*Serinus canaria*) have an intergenic region of similar length to that of ABV-062_CG_, they do not possess the uORF (2). A sequence comparison indicates that ABV-062_CG_ shows 65 to 70% and 64 to 65% identities with ABVs (genotypes 1, 2, 4, C1, and C2) and BDVs, respectively. The 3′ noncoding sequence of ABV-062_CG_ contains 54 nucleotides, which is similar to that of BDVs and other ABVs. However, the identity of the 3′ noncoding region to both ABV and BDV is <72%. Interestingly, the 5′ noncoding region of ABV-062_CG_ is longer (179 nucleotides) than that of any previously described ABVs or BDVs in the GenBank database. The alignment of the nucleotide sequences for each individual gene revealed that the N, X, P, M, G, and L genes of ABV-062_CG_ share 69 to 70, 72 to 74, 72, 75 to 77, 67 to 71, and 63 to 66% nucleotide identities with ABVs and 67 to 76, 72 to 79, 67 to 75, 74 to 79, 65 to 66, and 63 to 70% nucleotide identities with BDVs. Phylogenetic analysis based on the complete genome demonstrated that ABV-062_CG_ and canary genotypes form a cluster that is more closely related to BDVs than to ABVs.

### Nucleotide sequence accession number.

The genomic sequence of ABV-062_CG_ was submitted to the GenBank database under the accession no. KF578398.
